# Exogenous melatonin improves glutathione content, redox state and increases essential oil production in two *Salvia* species under drought stress

**DOI:** 10.1038/s41598-020-63986-6

**Published:** 2020-04-23

**Authors:** Siamak Shirani Bidabadi, Joshua VanderWeide, Paolo Sabbatini

**Affiliations:** 1 Department of Horticulture, College of Agriculture, Isfahan University of Technology, Isfahan, 84156-83111 Iran; 2 Department of Horticulture, Michigan State University, East Lansing, MI 48824 USA

**Keywords:** Plant sciences, Plant stress responses

## Abstract

This research was conducted to understand the influence of foliar applied melatonin (0, 50, 100, 150 and 200 μM) on two *Salvia* species (*Salvia nemorosa* L., and *Salvia reuterana* Boiss) under conditions of water stress. Water stress was applied using a reduced irrigation strategy based on re-watering at 80%, 60% and 40% of the field capacity (FC). Increasing water stress, while significantly enhancing malondialdehyde (MDA), H_2_O_2_, electrolyte leakage, oxidized glutathione (GSSG), and total glutathione (GT), reduced glutathione (GSH), catalase (CAT), peroxidase (POD), superoxide dismutase (SOD) and glutathione reductase (GR) activities, which led to a marked reduction in fluorescence (*Fv/Fm*). Foliar application of melatonin alleviated the oxidative stress by increasing GT, CAT, POD, SOD and GR activities and reducing GSSG. In particular, melatonin heightened GSH content as well as the ratio of GSH/GSSG when compared to non-sprayed water stressed plants. Melatonin-treated plants had significantly lower SOD and POD activities than control plants under drought stress, while the CAT activity was enhanced with the foliar treatment. Essential oil yield of both *Salvia* species increased with the decrease in irrigation from 80% to 60% FC but diminished with the more severe water deficit (40% FC). Essential oil components of *Salvia nemorosa* were β- caryophyllene, germacrene- B, spathulenol, and cis- β- farnesene, while (E) - β- ocimene, α- gurjnnene, germacrene-D, hexyl acetate and aromadendrene was the major constituents of *Salvia reuterana*. When plants were subjected to water deficit, melatonin treatment increased the concentration and composition of the essential oil. In particular, melatonin treatments improved the primary oil components in both species when compared to non-melatonin treated plants. In conclusion, reduced irrigation regimes as well as melatonin treatments resulted in a significant improvement of essential oil production and composition in both *Salvia* species.

## Introduction

Plants growing in arid and semi-arid regions often experience conditions of reduced precipitation and erratic rainfall patterns. Water stress reduces chlorophyll content and consequently, photosynthetic performance^1–3^. Severe water limitations can cause an imbalance between cellular redox components, where antioxidant defences do not counteract the greater production of reactive oxygen species (ROS). This induces a series of oxidative damages, leading to impaired growth and development^4,5^ and a reduction in plant fresh and dry weight^6^. Additionally, high ROS concentrations within particular organelles can oxidize cellular constituents, such as membrane lipids, indicated by an increase in the lipid peroxidation by-product malondialdehyde (MDA), as well as carbohydrates, proteins, and DNA^7^. Plants exhibit several physiological adaptations to deal with the negative impact of water stress^8^. In order to cope with oxidative stress, plants activate their antioxidant system and the major enzymes involved are superoxide dismutase (SOD), peroxidise (POD) and catalase (CAT)^9,10^. Maintenance of higher levels of antioxidants is a pivotal physiological strategy adopted by plants to counteract the negative effects of ROS^11^.

*Salvia* species are an important source of secondary metabolites that have relevant benefits to human nutrition and health. Leaves of *Salvia* plants have been used in traditional medicine for centuries and are also listed in the official pharmacopoeias^4^. *Salvia reuterana* and *Salvia nemorosa* are native *Salvia* plant species of Iran whose essential oils and chemical components have been studied extensively^12,13^. *Salvia reuterana* is mostly cultivated in the central highlands of Iran and possesses neurological, antimicrobial, antioxidant, chemotherapeutic and antidiabetic properties^13,14^. According to a study conducted by Rajabi *et al*.^14^, *Salvia reuterana* is present in the central regions of Iran with arid and semi-arid climate, while *Salvia nemorosa* is more likely to be originated from the northern regions of Iran with higher humidity and rainfall. Despite their importance as medicinal plants, *Salvia* are drought sensitive, and oxidative damages have previously been reported in drought-stressed *Salvia* plants, which were shown to activate the antioxidant system^4^.

Melatonin (N-acetyl-5-methoxytryptamine), a derivative of tryptophan, is ubiquitous in living organisms and is reported to have a hormone-like role in several plant species^15–17^. Melatonin was found to operate as a mammalian hormone and neurotransmitter, however its presence and potential role in plants was only recently documented^18–20^. In plants, melatonin is involved in multiple physiological processes, including growth and photosynthesis, biological rhythms, rooting and seed germination, and osmoregulation^21^. Its ability to protection against abiotic and biotic stresses, in particular low temperature^22^, drought^23,24^, UV-B^25^ and heavy metal pollution^26^ has also been documented. Many investigations have indicated that melatonin could be considered a plant growth regulator, with a mode of action that works in conjunction with other chorismate-derived phytohormones, including indole-3-acetic acid (IAA)^27^ and salicylic acid^28^. Melatonin has a pivotal role in the activation of the antioxidant system and induces changes in gene expression of several physiological mechanisms^21,29^.

*Salvia* species, important medicinal plants in arid and semi-arid regions, are often at risk of drought stress^12–14^. Between the two *Salvia* species reported on here, drought resistance in *Salvia reuterana* is known to be higher than *Salvia nemorosa*. In this contest, improvement in drought resistance in both *Salvia* species has an important practical significance for the agricultural production of medicinal herbs. Currently, there is no report on the potential use of exogenous melatonin in *Salvia* production. Therefore, the present study was conducted to investigate the impact of foliar melatonin on growth, photosynthetic rate, oxidative stress, and essential oil production under drought stress conditions of two *Salvia* species. The results would grant new insights into melatonin function in plants and provide a new tool for medicinal plant cultivation to consistently improve yield and quality.

## Material and methods

### Experimental design and treatments

*Salvia* species used in this study included *Salvia nemorosa* L., and *Salvia reuterana* Boiss. Commercial seedlings (15–20 cm height), purchased from a commercial nursery (Pakan Seed Institute of Isfahan, Iran), were transplanted (two per pot) into plastic pots (11 cm diameter and 8.5 cm height). The experiment was carried under controlled environment conditions in the experimental greenhouse at Isfahan University of Technology (Iran) during 2018. Environmental parameters were 20°–15 °C day-night temperature, 60% relative humidity and 16 h photoperiod with a constant average Photosynthetic Active Radiation (PAR) of 300 µmol m^−2^ s^−1^ as previously suggested by Caser *et al*.^30^. The study was conducted in a greenhouse with uniform environmental conditions; therefore, the experiment was arranged as a completely randomized design (RCD) with two factors (irrigation and foliar applied melatonin). Each treatment contained nine plant samples of three biological replicates. Each replicate consisted of a pot (three plants/pot). For each measurement, three samples were randomly separated from different parts of the plant. The experimental factors were irrigation treatments at 3 levels; irrigation to 80% field capacity (control), 60% field capacity (mild stress) and 40% field capacity (severe stress) according to the method reported by Sedaghat *et al*.^31^. The irrigation treatments were associated with foliar applied melatonin at five levels (0, 50, 100, 150 and 200 μM). Melatonin concentrations were selected based on the experiments reported by Zhang *et al*.^32^, in two *Salvia* species (*Salvia nemorosa* L., and *Salvia reuterana* Boiss). The melatonin (Sigma–Aldrich) solutions were prepared according to Li *et al*.^33^ by dissolving the solute in ethanol followed by dilution with phosphate buffered saline (PBS) to concentrations of 0, 50, 100, 150 and 200 μM. To improve uniformity of the spray application, for every 100 ml of solution two drops of surfactant (Twin 20) was added to the solution. Melatonin solutions were applied using a manual pump (30 ml per plant), two times per week for 45 days. The control treatment was sprayed with distilled water. The experiment was started after 15 days from the transplanting of the seedlings, after acclimation of the plants to the media and the greenhouse conditions. *Salvia* plants were grown in sandy loam soil according to Pourmeidani *et al*.^34^. To determine the amount of water needed for each irrigation regime, at the beginning of the experiment the soil field capacity (FC) was determined for each pot using the weighing method^34^. The collected data was used to determine the different amount of water to apply as percentage of the field capacity (FC). Four kg of soil was placed in the oven at 103 °C for 48 hours to determine soil dry weight. Afterwards, the pots were filled with the oven-dried soil and irrigated until water saturation. After 24 hours the pots were weighed every two hours. To avoid evaporation, the pot was covered with a plastic cover. The percentage of water in soil under FC condition was determined by the following equation:

$$percentage\cdot of\cdot water\cdot in\cdot the\cdot soil=(Soil\cdot fresh\cdot weight-Soil\cdot dry\cdot weight/Soil\cdot dry\cdot weight)\times 100$$

After deducting pot weight and weight of the dry soil, the amount of water stored in the FC condition was determined and irrigation treatments (80, 60 and 40% FC) were calculated accordingly. Therefore, pots were weighed daily based on each treatment and water was added to maintain the specific FC level in each pot. Two weeks after full flowering stage, 120-day old plants were harvested by cutting stems 5 cm above the soil surface and the measurements were made on freshly mature leaves. All the data were subjected to analysis of variance (ANOVA) and means were then separated using the Least Significant Difference (LSD) using SAS (version 8.2; SAS Institute, Cary, NC, USA) and MSTAT-C software^35^ (Freed and Scott 1989). The heatmap was generated using Heml Heatmap Illustrator Software^36^.

### Chlorophyll fluorescence (Fv/Fm) ratio and Electrolyte leakage (EL)

The maximum photochemical efficiency of photosystem II (*Fv/Fm*) was measured with a fluorometer (Walz, Effeltrich, Germany) after 30 min of leaf dark adaptation. The *Fv/Fm* ratio was calculated as: *Fv/Fm* = (*Fm- F0*)/*Fm*, where, Fm and F0 represented the maximum and minimum yields of dark-adapted leaves, respectively. Electrolyte leakage (EL) was measured by using a conductivity meter according to Ozden *et al*.^37^.

### Malondialdehyde (MDA) and Hydrogen peroxide (H_2_O_2_) concentrations

The accumulation of MDA because of lipid peroxidation was assessed by the Thiobarbituric acid (TBA) according to Wang *et al*.^27^ and was calculated on a fresh weight basis, using the following formula:

$$(nmol\cdot \cdot MDA\cdot {g}^{-1}\cdot FW)=6.54(OD532-OD600)-0.56(OD450)\times 1000$$

H_2_O_2_ was assessed spectrophotometrically after the reaction with Potassium iodide (KI), according to the method reported in Velikova and Loreto (2005)^38^. The content of H_2_O_2_ was calculated using a standard curve with known concentrations of hydrogen peroxide.

### Glutathione pool estimation

Total glutathione was measured according to the method described by Sahoo *et al*.^39^. The fresh leaf samples were centrifuged at 11500 × g for 15 min at 4 °C and the 0.4 mL of the supernatant was then added to 1 mL of 0.5 M potassium phosphate buffered at pH 7.5. Then, 100 µL 2-nitrobenzoic acid (DTNB, 10 mM), 200 µL bovine serum albumin (BSA, 10 mM), 100 µL nicotinamide adenine dinucleotide (NADH, 0.5 mM) were added to the vial and incubated at 37 °C for 15 min. Finally, the mixture was allowed to cool and the change in absorbance at 412 nm was measured against a blank containing 0.4 mL of water. The results were expressed as nmol per gram fresh weight (nmol *gFW*^−1^). For the GSSG assay, the GSH was removed by addition of 2-vinylpyridine to the supernatant for 1 h at 25 °C. The extract (100 µL) was mixed with 600 µL reaction buffer (100 mM potassium phosphate buffer containing 5 mM EDTA, pH 7.5), 100 µL of diluted yeast glutathione reductase (GR, 20 U/mL), and 100 µL of 10 mM DNTB. The reaction was initiated by adding 100 µL of 2.5 mM nicotinamide adenine dinucleotide phosphate (NADPH), and after mixing thoroughly, the rate of absorption change at 412 nm was measured spectrophotometrically. The results were expressed as nmol per gram fresh weight (nmol *g FW*^−1^). GSH content was calculated by subtracting GSSG from total glutathione.

### Enzyme (CAT, POD, SOD and GR) extraction and assay

Fresh foliar tissue (0.2 g) from *Salvia* seedlings (uppermost leaves) was harvested, weighed, washed with distilled water and then homogenized with a mortar and pestle with 5 ml chilled sodium phosphate buffer (50 mM, pH 7.8). The homogenates were centrifuged at 15,000 g for 15 min at 4 °C. The supernatant was stored at 4 °C and used for CAT, POD, SOD and GR assays. CAT activity was measured by the method of Blume and McClure (1980). CAT activity was expressed as μmol of hydrogen peroxide oxidized per minute per milligram of protein. POD activity was determined spectrophotometrically, by measuring the oxidation of o-dianisidine (3, 3- dimethoxybenzidine) at 460 nm as described by Ranieri *et al*.^40^ and expressed as units (μmol of dianisidine oxidized per minute) per mg of protein. SOD activity was estimated by recording the decrease in absorbance of superoxide-nitroblue tetrazolium complex by the enzyme (Cavalcanti *et al*.)^41^. A 3 mL of reaction mixture was prepared with 0.1 mL of 13 mM L-methionine, 0.1 mL of 75 µM *p*- nitroblue terazolium chloride (NBT), 0.1 mL of 100 µM EDTA, 0.1 mL riboflavin (2 µM) in a 1.5 mL of 50 mM potassium phosphate buffer pH 7.8, 50 µL of the enzymatic extract and distilled water. The reaction was started under illumination of fluorescent lamp (30 W) at 25 °C and stopped 5 min later by turning the lamp off. The blue formazane produced by NBT photo-reduction was measured as an increase in absorbance at 560 nm. The control reaction mixture had no enzyme extract (with maximal colour formation). The blank solution had the same complete reaction mixture, but was kept in the dark. One SOD unit was defined as the amount of enzyme required to inhibit 50% of the NBT photo-reduction in comparison with tubes lacking the plant extract and expressed as a unit of enzyme activity per mg of protein. Glutathione reductase (GR) activity was identified by following the rate of NADPH oxidation at 340 nm according to Balabusta *et al*.^42^. The assay mixture included 0.5 mM NADPH, 10 mM GSSG, 6.25 mM MgCl_2_ in 0.1 M phosphate buffer (pH 7.5), and 100 µL of the enzyme extract in the total volume of 400 µL. GR activity was expressed as µmol of NADPH oxidized during 1 min per 1 mg of proteins (µ mol min^−1^ mg protein^−1^). Protein content of the extracts was determined according to the method of Bradford (1976)^43^.

### Oil extraction and chemical composition

Extraction and yield calculation of essential oil in *Salvia nemorosa* L., and *Salvia reuterana* Boiss., was achieved according to the method of Fernandes *et al*.^44^ using 120 days old plants (from seed germination to full bloom stage). Plant tissue collected from the flowering stems of *Salvia nemorosa* L., and *Salvia reuterana* Boiss., were dried in a stove at 30 °C until constant weight was obtained, then crushed into small fragments by a grinding machine and stored in a freezer at −20 °C until the beginning of the distillation process. The essential oils were extracted by hydro-distillation in a Clevenger-type apparatus over 3 h in 1.5 L of water. Water was then removed from the essential oil using anhydrous sodium sulphate and then weighed. The essential oil content was determined based on the volume extracted per 100 g of leaf dry biomass (% w/v). The essential oil yield was also determined by multiplying the essential oil content by the leaf dry biomass (g plant^−1^).

### GC/MS conditions and analysis and identification of volatile components

The composition of essential oils of *Salvia nemorosa* L. and *Salvia reuterana* Boiss was detected using a gas chromatograph attached to a mass spectrometer (tandem GC-MS), including a Shimadzu GC-9 gas chromatograph equipped with a DB-5 (dimethylsiloxane, 5% phenyl) fused silica column (30 m × 0.25 mm × 0.25 m), with internal diameter of the column 0.25 mm, film thickness of 0.25 µm. Helium was used as the carrier gas at a flow rate of 2 ml/min with a linear velocity of 32 cm/s. The flame ionization detector (FID) temperature was 265 °C and the injector temperature was 250 °C. The percentage of the different compounds was calculated by the area normalization method, without considering response factors. Identification of the components was based on a comparison of their mass spectra (MS) with a computer library or with the authentic compound (standard spectra) and confirmation of compound identities was also obtained using relative retention indices (RI). Moreover, the percentage of each component of the essential oil was calculated by utilizing the peak height as well as the peak area.

## Results and discussion

### Analysis of main and interaction effects

**Figure 1:**
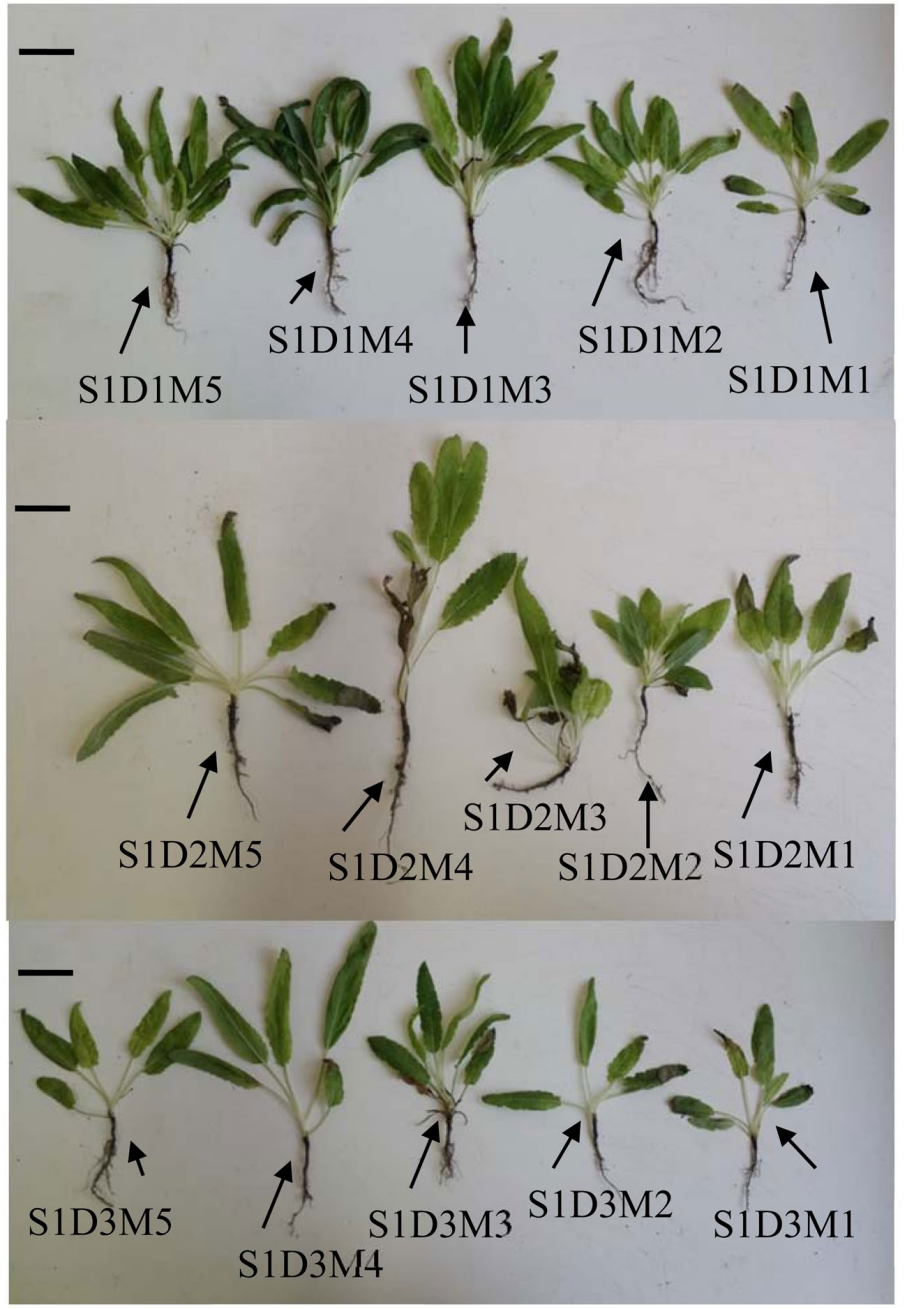
Schematic overview of *Salvia nemorosa* L, (indicated as S1) responses to three irrigation regimes containing: D1, D2 and D3 (80%, 60% and 40% FC, respectively), with melatonin levels including: M1, M2, M3, M4 and M5 (0, 50, 100, 150 and 200 µM, respectively). Horizontal bar = 1 cm.

Our study evaluated the responses of *Salvia nemorosa* L. and *Salvia reuterana* Boiss. to foliar melatonin under reduced irrigation; in conditions of water stress (Fig. 1). The results reported significant main and interactive effects (at 5% and 1% probability levels) of species, water stress, melatonin, species × water stress, species × melatonin, water stress × melatonin and species × water stress × melatonin on electrolyte leakage (EL), essential oil content (EOC), essential oil yield (EOY), oxidized glutathione (GSSG), total glutathione (GT), reduced glutathione (GSH), glutathione reductase (GR), and superoxide dismutase (SOD) (Tables 1 and 2). *Fv/Fm*, MDA, H_2_O_2_, GSH/GSSG, CAT and POD activity showed significant change in response to all main and interactive effects other than the interactive effect of species × water stress × melatonin (Tables 1 and 2). Interactive effect of water stress × melatonin was significant on all the above-mentioned traits at 1% probability levels (Tables 1 and 2). The interactive effect of species × melatonin was regarded non-significant only in terms of MDA accumulation, GR and POD activities (Tables 1 and 2). However, interactive effects of species × melatonin and species × water stress on GSH/GSSG ratio and CAT activity showed no significant differences (Tables 1 and 2). Similar effect of species, melatonin, water stress, and species × water stress significantly triggered different responses in *Salvia* species were recently reported^14,45^. The significant main and interactive effects of species, water stress and species × water stress in the *Salvia* cultivars studied in our experiment were also in line with the results obtained in another *Lamiaceae* species, *Thymus*^44^, further supporting these results.

**Table 1:** Mean square values of the analysis of variance of electrolyte leakage (EL%), *Fv/Fm* ratio, lipid peroxidation indicated by malondialdehyde (MDA) (nmol g^−1^), H_2_O_2_ content (nmole g^−1^), essential oil content (EOC %) and essential oil yield (EOY g plant^−1^).

Source of variants	df	EL	*Fv/Fm*	MDA	H_2_O_2_	EOC	EOY
Species (S)	1	230.40^**^	0.03^**^	9.29^**^	181.62^**^	0.35^**^	0.08^**^
Drought (D)	2	6910.24^**^	0.72^**^	260.72^**^	10699.09^**^	0.52^**^	0.13^**^
Melatonin (M)	4	177.52^**^	0.02^**^	1.92^**^	326.68^**^	0.24^**^	0.06^**^
S × D	2	36.66^**^	0.002^**^	1.43^**^	13.80^**^	0.07^**^	0.02
S × M	4	3.39^**^	0.0006^**^	0.02 ^ns^	2.67^**^	0.003^*^	0.0008^**^
D × M	8	33.85^**^	0.0008^**^	0.28^**^	37.63^**^	0.01^**^	0.003^**^
S × D × M	8	3.46^**^	0.0002^ns^	0.03^ns^	1.17^ns^	0.003^**^	0.0008^*^
Error	60	0.81	0.00009	0.06	0.67	0.0008	0.0002
CV (%)		5.20	1.62	6.31	2.30	4.21	4.19

^*^ and **: Significant at the 5% and 1% probability levels, respectively., ns: Non- Significant, according to the LSD multiple range test at P ≤ 0.05. df, degrees of freedom.

**Table 2:** Mean square values of the analysis of variance of oxidized glutathione (GSSG) (nmole g^−1^ FW), total glutathione (GT) (nmole g^−1^ FW), reduced glutathione (GSH) (nmole g^−1^ FW), GSH/GSSG, Glutathione reductase (GR) (µmol min^−1^ mg protein^−1^), catalase (CAT) activity (U mg^−1^ protein), peroxidase (POD) activity (U mg^−1^ protein) and superoxide dismutase (SOD) and activity (U mg^−1^ protein).

Source of variants	df	GSSG	GT	GSH	GSH/GSSG	GR	CAT	POD	SOD
Species (S)	1	85.71^**^	30731.13^**^	36352.05^**^	31.75^ns^	0.0004^**^	0.0004^**^	0.004^**^	0.007^**^
Drought (D)	2	20875.64^**^	521364.43^**^	188251.46^**^	33110.52^**^	140.29^**^	0.02^**^	0.63^**^	0.004^**^
Melatonin (M)	4	281.50^**^	40178.05^**^	53509.01^**^	1092.27^**^	3.49^**^	0.0008^**^	0.01^**^	0.001^**^
S × D	2	63.56^**^	2517.30^**^	3992.04^**^	15.44^ns^	0.47^**^	0.000003 ^ns^	0.0004^*^	0.003^**^
S × M	4	8.28^**^	1072.17^**^	806.11^**^	6.62^ns^	0.01 ^ns^	0.00006 ^ns^	0.00008 ^ns^	0.0002^**^
D × M	8	80.54^**^	6338.33^**^	9166.92^**^	345.67^**^	0.08^**^	0.00008^*^	0.003^**^	0.0001^**^
S × D × M	8	3.88^**^	418.35^*^	346.75^*^	4.54^ns^	0.05^*^	0.00005^ns^	0.00006^ns^	0.0003^**^
Error	60	0.6	158.12	190.19	11.76	0.02	0.00003	0.00008	0.000002
CV (%)		2.98	3.80	4.95	11.62	3.42	10.87	6.23	4.74

* and **: Significant at the 5% and 1% probability levels, respectively., ^ns^: Non- Significant, according to the LSD multiple range test at P ≤ 0.05. df, degrees of freedom.

### Chloroplast membrane integrity and photosynthetic efficiency

**Figure 2:**
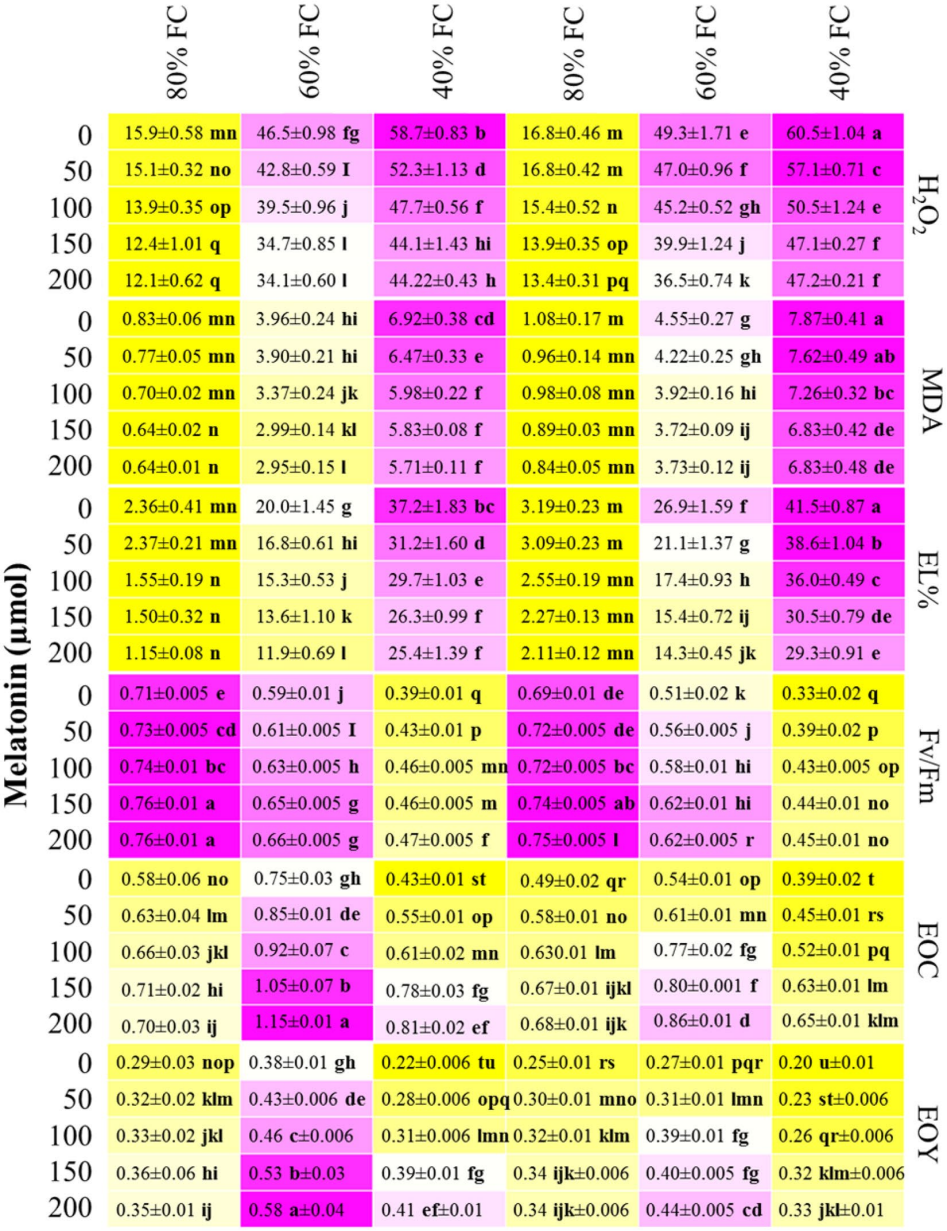
Heatmap representation of the interactive effect of drought stress and melatonin application on H_2_O_2_ content (nmol g^−1^), lipid peroxidation indicated by malondialdehyde (MDA) (nmol g^−1^), electrolyte leakage (EL%), *Fv/Fm* ratio, essential oil content (EOC %), and essential oil yield (EOY) (g plant^−1^) of *Salvia nemorosa* L., and *Salvia reuterana* Boiss. Three-way ANOVA test: Means followed by the same letter are not significantly different by the LSD Multiple Range test at P ≤ 0.05. Purple and yellow represent increased and decreased values, respectively.

Chloroplasts are a primary location for the generation of ROS in plant cells and they have been reported to be the most sensitive organelles to abiotic stress^46–48^. The accumulation of ROS causes damage to chloroplast being that the chloroplasts are the major source of activated O_2_ in plants^26^. Water stress has been reported to be responsible for generating severe oxidative stress in plant crops, as indicated by an increase in H_2_O_2_ and lipid peroxidation levels^48,49^. This was observed here, where both *Salvia* species grown under water stress exhibited a significant increase in H_2_O_2_ generation (Fig. 2). However, melatonin treatments significantly compensated for oxidative damage caused by the water stress in both *Salvia nemorosa* and *Salvia reuterana* (Fig. 2). The protective role of melatonin may be due to its effect in maintaining a steady state of intracellular ROS concentrations, thereby reducing membrane damage from drought stress^50^. Our results are consistent with those reported in other studies using *Cucumis sativus* L. seedlings^19^ and grapevines^50^ under water and salinity stress. Lipid peroxidation expressed as MDA accumulation is a good representation of the oxidative damage in plants. Therefore, the increased MDA production as a result of water stress (Fig. 2) can be considered as evidence of oxidative plant stress leading to cell production of reactive oxygen species (ROS) such as H_2_O_2_ generation^51^. This was further evidenced by the increase in electrolyte leakage in response to increasing deficit irrigation (Fig. 2). Similar to H_2_O_2_, melatonin treatment had a significant effect on mitigating MDA production and EL in both *Salvia* species (Fig. 2). Meng *et al*.^50^ reported that under water stress conditions, grapevines leaves of melatonin-treated cuttings accumulated less MDA and had lower relative electrolytic leakage than cuttings that did not receive any melatonin treatment. As expected, the negative effects of mild and severe water stress (60 and 40% FC, respectively) on the quantum efficiency of Photosystem II (*Fv/Fm*) were significantly reduced in *Salvia reuterana* when compared to *Salvia nemorosa* (Fig. 2). These results are consistent with Huang *et al*.^52^ and Ahmad *et al*.^53^ who reported that melatonin application alleviated the negative effects of oxidative stress and ROS production. Even at mild water stress (60% FC), a reduced *Fv/Fm* ratio was recorded in the two species, and a more intense water stress (40% FC) had a further negative effect on this physiological parameter (Fig. 2). However, both *Salvia* species sprayed with melatonin reported a positive response, with an improved ratio of *Fv/Fm* under water stress (Fig. 2). In accordance with the results reported by Wang *et al*.^23^, melatonin has the potential to maintain high photosynthetic efficiency in plants and our results proved that melatonin application relieved water stress improving the efficiency of *Fv/Fm* (Fig. 2). In support of our findings, melatonin has been already reported that improves electron transport in chloroplast under different environmental stresses^51,54^ and consequently photosynthetic efficiency (*Fv/Fm*). Melatonin has the potential to protect plants from the adverse effects of drought stress by enhancing the ROS scavenging efficiency. It helps in protection of photosynthetic apparatus and reduction of drought induced oxidative stress^53^. These results demonstrated that foliar application of melatonin improved photosynthetic capacity and reduced the oxidative damage in *Salvia* species under water stress conditions, and supported the hypothesis that exogenous application of melatonin could effectively enhance stress tolerance in plants^54,55^.

### Glutathione pool and enzymatic antioxidant activities

**Figure 3:**
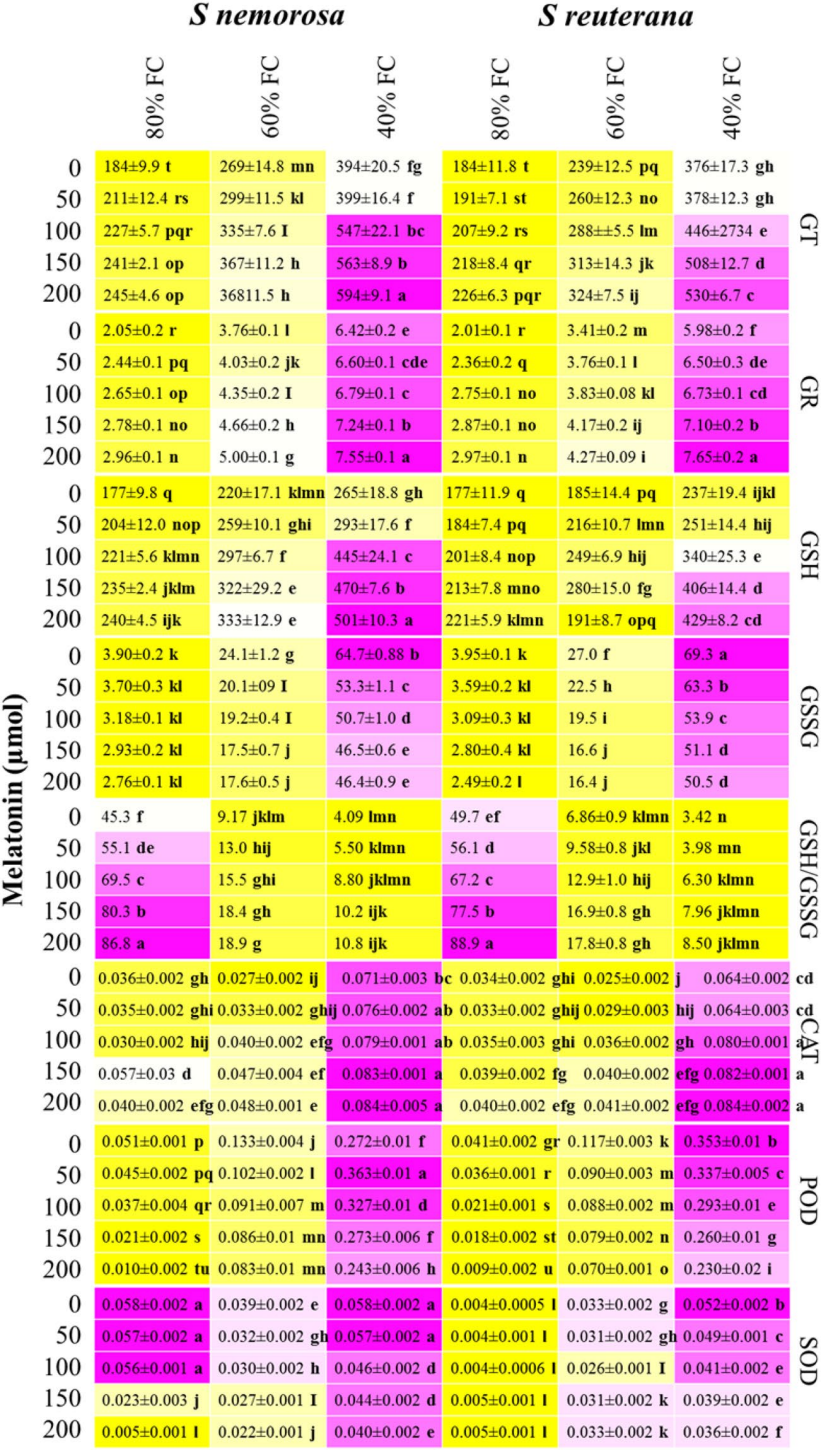
Heatmap representation of the interactive effects of drought stress and melatonin application on total glutathione (GT), glutathione reductase (GR), reduced glutathione (GSH), oxidized glutathione (GSSG), GSH/GSSG, catalase (CAT) activity (µ mole min^−1^ mg protein), peroxidase (POD) activity (µ mole min^−1^ mg protein), and superoxide dismutase (SOD) activity (µ mole min^−1^ mg protein) of *Salvia nemorosa* L., and *Salvia reuterana* Boiss. Three-way ANOVA test: Means followed by the same letter are not significantly different by the LSD Multiple Range test at P ≤ 0.05. Purple and yellow represent increased and decreased values, respectively.

When exposed to oxidative stress, plants can activate their oxygen scavenging systems by increasing the activity of antioxidant enzymes or by mobilization of their non-enzymatic antioxidants, such as reduced glutathione (GSH)^42,55^. In this study, both non-enzymatic and enzymatic antioxidants were evaluated. Glutathione Reductase (GR) exists primarily in the chloroplast and reduces glutathione disulfide (GSSG) to glutathione (GSH). GSH participates in enzymatic and non-enzymatic H_2_O_2_ degradation to maintain a reductive state within the cell^39^ and ameliorates ROS-induced damage. This was recently shown by Hasanuzzaman *et al*.^48,49^ in *Brassica napus*. Here, increasing drought stress produced higher cell H_2_O_2_ concentrations, but was mitigated by increasing melatonin concentration (Fig. 1). In response, GT, GR, and GSH were similarly modulated, reaffirming this the role of this system in ameliorating drought stress. A similar effect of melatonin on total glutathione content was observed also by Wang *et al*.^23^ on apple leaves when treated with a foliar melatonin application. Additionally, oxidized glutathione (GSSG) reported a significant increase when both *Salvia* species were subjected to water stress, but decreased with increasing melatonin (Fig. 3). The GSH/GSSG ratio is considered as an indirect indicator of oxidative stress and damage. When plants are exposed to oxidative stress GSSG is accumulated and the ratio of GSH to GSSG decreases^42,55^. In our study, mild and severe water stress decreased the GSH/GSSG ratio in both *Salvia* species, indicative of stress (Fig. 3). However, the increasing ratio of GSH/GSSG in response to exogenously applied melatonin showed the beneficial effect of melatonin in reducing oxidative stress and improving water stress tolerance, in line with the research reported by Galano *et al*.^29^ that also showed that foliar application of melatonin in wheat plants exposed to stress conditions elevated total GSH content, as well as significantly enhanced the ratio of GSH/GSSG compared to control plants and stressed plants without melatonin. Similar to glutathione, enzymatic antioxidant activity in plants subjected to increasing water stress regimes was enhanced, with the exception of SOD in *S nemorosa*. SOD, POD and CAT levels indicate that the main protective enzymes in the enzymatic defence system, can effectively scavenge reactive oxygen species^19,20^. Jafari *et al*.^14^ suggested the higher activity of SOD in medicinal plants was due to their increased resistant to drought stress, suggesting that *Salvia nemorosa* with higher SOD activity (0.058) could be more water stress tolerant than *Salvia reuterana* (0.052), as reported in Fig. 3. Additionally, the activity of catalase (CAT) activity increased with higher levels of melatonin application, whereas POD and SOD saw an opposite response in most cases (Fig. 3). Catalase is a member of the haem peroxidase family of enzymes that are distinctly responsible for the dismutation of H_2_O_2_ into H_2_O and O_2_^6^. Given this, and that POD and SOD have other roles within the redox system, it is understandable how only CAT activity increased similar to that of H_2_O_2_. Interestingly, Munne-Bosch *et al*.^4^ found that in response to the oxidative burst (H_2_O_2_) marking the start of the ripening process in guava, MDA concentration, POD, and SOD activity increased, whereas, GT, GR, GSH, and CAT decreased. These results suggest that CAT operates similarly to GR in response to oxidative stimuli, but are not related with POD and SOD activity, a result seen here in response to the redox-modulator melatonin.

### Oil yield and chemical composition

Mild water stress had a similar impact on both EOC and EOY, with 60% FC having the highest values, and 80% FC, the lowest. Previous research has reported a decrease in essential oil content in different *Lamiaceae* species under water stress^45^, while others have shown an increase in essential oil content in *Lamiaceae* species under drought conditions^56^. As seen here, cultivar-specific responses to water stress may factor into the differences observed by these studies. Likewise, increasing levels of foliar melatonin application had a positive impact on EOC and EOY in both *Salvia* species (Fig. 2). Taken together, the highest essential oil content (1.15%) and essential oil yield (0.58%) were recorded with 200 µM melatonin applied on *Salvia nemorosa* grown under mild water stress (60% FC) conditions (Fig. 2). Despite the clear effect of both water stress and melatonin, the impact of water was greatest. This is displayed by the 60% FC treatment with 0 µM melatonin having a greater EOC and EOY than the other irrigation treatments at 200 µM melatonin for *Salvia nemorosa*. The composition of essential oil produced under different water stress regimes and melatonin treatments are reported in Tables 3 and 4. Eighteen compounds, accounting for 81.87%-98.87% of the total essential oil content, were identified in *Salvia nemorosa* (Table 3). The main essential oil components of *Salvia nemorosa* were β- caryophyllene (37.53–40.13%), germacrene- B (19.83–21.37%), spathulenol (6.37–8.72%), and cis- β- farnesene (5.75–7.83%) (Table 3). Evaluation of essential oil yields of *Salvia reuterana* also resulted in fourteen components representing 81.34–98.46% of the total essential oil content. Among the characterized compounds, (E) - β- ocimene (35.76–38.82%), α- gurjnnene (16.23–17.66%), germacrene-D (10.7–13.15%), hexyl acetate (6.55–9.21%), and aromadendrene (3.33–5.65%) were the major constituents (Table 4). Those results demonstrated that the highest percentage of major essential oil constituents were observed when both *Salvia nemorosa* and *Salvia reuterana* were exposed to mild water stress (60% FC) compared to those that were under severe water stress (40% FC). The various levels of melatonin affected the constituents of essential oils of both *Salvia* species in different ways. Treatment of *Salvia nemorosa* with 150 µM of melatonin resulted in the accumulation of β- caryophyllene, Germacrene- B, spathulenol and cis- β- farnesene to a significant extend and more than the other treatments (Table 3), while for *Salvia reuterana*, 150 µM of melatonin enhanced the accumulation of hexyl acetate and aromadendrene and 200 µM of melatonin caused an increase in (E) - β- Ocimene, α- gurjnnene and germacrene-D more than other treatments (Table 4). Similarly, Sarrou *et al*.^57^ also observed that melatonin treated lemon balm (*Melissa officinalis*) reported an increase in several compounds, in particular nerol, a result similar to our study. Although the role of melatonin in the biosynthesis of essential oil in medicinal plants has not been well understood, one of the mechanisms proposed is related to the similarity in plant function and chemical structure between indole-3-acetic acid (IAA) and melatonin^20,24,28^, which are both derived from chorismate^28^. It has been reported that IAA promotes essential oil synthesis in several medicinal plants^58,59^, and given that melatonin has an auxin-like (IAA) activity, it would be expected that melatonin also promotes the synthesis of essential oils. It has been hypothesized that the increased amount of essential oil of *Salvia* species in response to foliar melatonin application can be attributed to the potential enhancement of meristematic cells, site of production of several chemical compounds that are fundamental for oil biosynthesis^58,59^.

**Table 3:** Essential oil compound of *Salvia nemorosa* L., under different irrigation regimes and melatonin treatments.

Compound	S1D1M1	S1D1M2	S1D1M3	S1D1M4	S1D1M5	S1D2M1	S1D2M2	S1D2M3	S1D2M4	S1D2M5	S1D3M1	S1D3M2	S1D3M3	S1D3M4	S1D3M5	RI^a^
α-Pinene	TR	TR	TR	TR	TR	0.04	0.04	0.06	0.08	TR	TR	TR	TR	TR	TR	987
Sabinene	0.21	0.19	0.27	0.29	0.28	0.56	0.59	0.64	0.74	0.81	TR	0.04	0.06	0.06	0.06	1006
**β-Caryophyllene**	**37.53**	**37.88**	**37.69**	**38.45**	**38.61**	**39.12**	**39.79**	**39.96**	**40.13**	**39.87**	**36.68**	**37.54**	**38.81**	**38.96**	**39.92**	**1011**
α-Terpinene	TR	TR	TR	TR	TR	1.4	1.5	1.59	0.12	0.07	TR	TR	TR	TR	TR	1017
Camphor	0.03	0.03	0.05	0.04	0.08	0.08	0.07	0.05	0.08	0.09	TR	TR	TR	TR	TR	1025
Hexyl butyrate	0.21	0.40	0.43	0.76	0.32	0.64	0.82	0.81	0.86	0.75	TR	TR	0.06	0.07	0.06	1033
Borneol	1.4	1.6	1.2	1.7	1.3	1.9	2.00	2.13	2.39	1.86	1.80	2.1	3.3	3.4	2.4	1039
Pulegone	1.37	1.73	1.94	1.91	1.82	1.46	1.48	1.55	1.83	2.08	1.59	1.78	2.32	2.64	1.58	1046
β-Thujone	0.7	1.13	1.54	1.53	1.66	1.64	1.83	1.46	1.96	1.95	1.35	1.11	2.37	2.35	2.39	1050
Nerolidol	0.78	0.85	0.84	0.85	0.89	1.21	1.42	1.47	1.51	1.88	1.22	1.31	2.27	2.33	2.32	1057
Valencene	3.23	3.65	3.54	3.63	3.68	2.91	2.98	3.17	3.27	3.58	3.09	3.57	4.84	4.95	5.93	1066
**Spathulenol**	**6.37**	**7.18**	**7.45**	**7.58**	**7.74**	**8.12**	**8.35**	**8.65**	**8.72**	**8.47**	**5.03**	**6.11**	**7.27**	**7.56**	**7.77**	**1084**
**cis- β -Farnesene**	**5.75**	**5.68**	**5.82**	**5.85**	**5.92**	**6.21**	**6.48**	**6.77**	**6.85**	**6.41**	**5.29**	**6.55**	**7.69**	**7.83**	**7.80**	**1091**
**Germacrene-B**	**19.83**	**19.90**	**20.19**	**20.60**	**20.57**	**20.84**	**20.85**	**21.11**	**21.37**	**20.66**	**17.97**	**18.65**	**19.88**	**20.04**	**19.75**	**1102**
Germacrene-D	1.66	1.84	2.31	2.54	2.72	2.28	2.74	2.81	2.81	2.39	1.85	1.08	2.14	2.13	2.16	1125
(E)-β-Ocimene	0.14	0.53	0.45	0.62	1.31	1.26	1.23	1.56	1.71	1.67	1.11	0.89	TR	TR	TR	1148
Limonene	0.21	0.17	0.75	0.94	0.85	1.16	1.19	1.26	1.66	1.85	1.49	1.23	1.00	1.00	0.56	1159
Valencene	2.45	2.69	3.15	2.94	2.87	2.91	2.15	2.55	2.78	2.91	1.42	2.71	2.44	1.84	1.93	1165
TCC	81.87	85.45	87.62	90.23	90.62	93.74	95.51	97.60	98.87	97.30	79.89	84.67	94.45	95.16	94.63	

S1: Salvia nemorosa L. D1: irrigation at 80% Fc, D2: irrigation at 60% Fc, D3: irrigation at 40% Fc, M1 (0 µM melatonin), M2 (50 µM melatonin). M3 (100 µM melatonin), M4 (150 µM melatonin) and M5 (200 µM melatonin).

^a^The data were sorted based on the Retention Index (RI) of components.

TR = trace, less than 0.01%., TCC = Total chemical composition

**Table 4:** Essential oil compound of *Salvia reuterana* Boiss., under different irrigation regimes and melatonin treatments.

Compound	S2D1M1	S2D1M2	S2D1M3	S2D1M4	S2D1M5	S2D2M1	S2D2M2	S2D2M3	S2D2M4	S2D2M5	S2D3M1	S2D3M2	S2D3M3	S2D3M4	S2D3M5	RI^a^
α-Copaene	0.60	1.1	0.50	0.70	0.90	1.30	1.40	1.54	1.87	1.74	TR	0.01	0.07	0.04	TR	961
Hexyl pentanoate	1.9	2.2	1.9	1.8	2.5	2.5	1.9	1.7	TR	0.1	0.1	0.3	0.42	0.45	0.76	984
**Hexyl acetate**	**6.55**	**6.61**	**6.78**	**6.89**	**6.95**	**8.19**	**8.70**	**8.94**	**9.21**	**8.88**	**6.04**	**7.14**	**7.65**	**7.88**	**7.73**	**1006**
Isopenthyl isovalerate	1.83	2.32	2.12	1.74	1.71	2.45	2.8	2.11	2.73	2.05	1.21	1.64	1.61	1.57	1.57	1011
Germacrene-B	0.33	0.43	0.55	0.64	0.68	0.58	0.74	0.59	0.68	0.89	TR	TR	TR	0.07	TR	1015
**(E)-β-Ocimene**	**35.76**	**35.77**	**35.74**	**35.93**	**35.70**	**38.61**	**38.79**	**38.41**	**38.68**	**38.82**	**37.12**	**37.98**	**37.77**	**37.61**	**37.85**	**1023**
Longifolene	0.45	0.46	0.42	0.53	0.53	0.79	1.60	1.74	1.79	1.82	TR	TR	0.05	0.12	TR	1029
β-Eudesmol	TR	TR	0.09	0.32	0.26	1.42	1.43	1.56	1.78	1.88	TR	TR	TR	0.07	TR	1033
**Germacrene-D**	**10.7**	**10.10**	**10.53**	**10.58**	**10.16**	**12.65**	**12.79**	**12.12**	**12.46**	**13.15**	**10.15**	**10.15**	**10.37**	**10.58**	**10.77**	**1046**
α -Farnesene	2.43	2.57	2.76	2.89	2.95	3.20	3. 20	3.18	3.36	3.28	TR	1.33	1.42	1.40	1.39	1051
Hexyl butyrate	1.23	1.28	1.45	1.67	1.82	2.42	2.64	2.86	2.97	2.88	2.09	1.07	1.69	1.59	1.64	1073
**α -Gurjunene**	**16.23**	**16.42**	**16.73**	**16.75**	**16.82**	**18.60**	**18.84**	**17.19**	**17.25**	**17.66**	**16.21**	**15.81**	**15.88**	**15.93**	**15.87**	**1080**
**Aromadendrene**	**3.33**	**3.68**	**3.58**	**3.85**	**3.93**	**5.14**	**5.17**	**5.15**	**5.65**	**5.31**	**4.03**	**4.10**	**4.21**	**4.35**	**4.12**	**1087**
β -Caryophyllene	TR	TR	TR	TR	TR	TR	TR	TR	TR	TR	TR	TR	TR	TR	TR	1120
TCC	81.34	82.94	83.15	84.29	84.91	97.85	96.8	97.09	98.43	98.46	76.95	79.53	81.14	81.66	81.70	

S2:Salvia reuterana Boiss. D1: irrigation at 80% Fc, D2: irrigation at 60% Fc, D3: irrigation at 40% Fc, M1 (0 µM melatonin), M2 (50 µM melatonin). M3 (100 µM melatonin), M4 (150 µM melatonin) and M5 (200 µM melatonin).

^a^The data were sorted based on the Retention Index (RI) of components.

TR = trace, less than 0.01%., TCC = Total chemical composition

### Hierarchical cluster analysis

**Figure 4:**
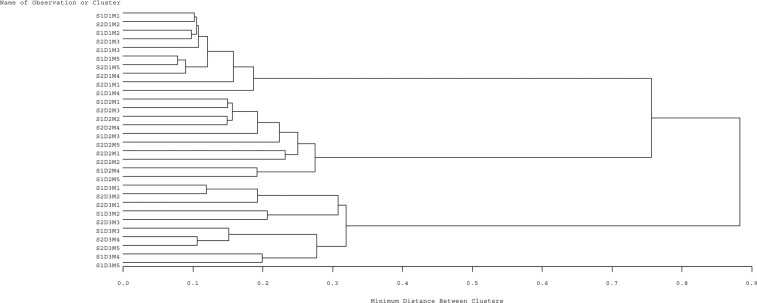
Hierarchical cluster analysis of exogenous melatonin in two *Salvia* species under reduced irrigation regimes, based on enzymatic and non-enzymatic antioxidant properties and essential oil yield. S1: *Salvia nemorosa* L., and S2: *Salvia reuterana* Boiss. D_1_: irrigation at 80% Fc, D_2_: irrigation at 60% FC, D_3_: irrigation at 40% Fc, M_1_ (0 µM melatonin), M_2_ (50 µM melatonin). M_3_ (100 µM melatonin), M_4_ (150 µM melatonin) and M5 (200 µM melatonin).

Cluster analysis of the combined treatments (foliar applied melatonin and irrigation regimes) is reported in Fig. 4, based on similarity. The cluster analysis was conducted in relation to enzymatic and non-enzymatic antioxidant properties and essential oil yield of the two *Salvia* species, which divided the 30 treatment combinations into 3 major clusters. Cluster I includes different foliar melatonin treatments for both *Salvia nemorosa* and *Salvia reuterana* under the normal irrigation regime (D_1_). Cluster II contains *Salvia nemorosa* and *Salvia reuterana* and foliar application of melatonin under mild drought stress (D_2_). Cluster III shows the foliar treatments of melatonin for *Salvia nemorosa* and *Salvia reuterana* grown under severe water stress (D_3_). These results demonstrated that the effect of water irrigation regimes was more prominent in modifying the physiological responses of the two *Salvia* species, followed as second level of importance by the impact of the foliar applied melatonin and lastly as third level of importance the genetic diversity of the two species of *Salvia* utilized in the research (Fig. 4). The cluster analysis suggests that irrigation is the best tool for controlling essential oil production in *Salvia*, but that the effect of melatonin was also great enough to induce similar responses to two *Salvia* cultivars differing in their response to water stress.

## Conclusion

The treatment of two *Salvia* cultivars with melatonin improved their antioxidant defence system against drought stress. This consequently enabled plants to increase their production of essential oil, especially under mild drought stress conditions. A particular effect of melatonin was observed on CAT activity compared with other antioxidant enzymes, which warrants further research in order to understand their role in increasing essential oil production in *Salvia* plants. Irrigation had the greatest impact on essential oil production in two *Salvia* cultivars, however, melatonin also had a significant added effect.
